# Life satisfaction, loneliness, and mental health in older adults: sociodemographic influences in a cross-sectional analysis

**DOI:** 10.1186/s12877-026-07671-9

**Published:** 2026-05-20

**Authors:** María González-Álamos, Manuel Torres, Blanca Bartolomé, Luis Vivanco

**Affiliations:** 1 Center for Biomedical Research of La Rioja (CIBIR), Rioja Health Foundation (FRS), Logroño, 26006 Spain; 2 UNESCO Regional Centre of Documentation and Information in Bioethics, Rioja Health Foundation (FRS), Logroño, 26006 España; 3 Faculty of Health Sciences, International University of La Rioja, Logroño, 26006 Spain; 4 University of La Rioja, Logroño, 26006 Spain; 5 Faculty of Medicine, University of Navarra, Pamplona, 31009 Spain

**Keywords:** Anxiety, Depression, Loneliness, Cohabitation, Marital status

## Abstract

**Background:**

Loneliness is a multidimensional experience that becomes particularly salient in older adulthood and is closely linked to depression and anxiety. Life satisfaction represents the cognitive component of subjective well‑being and may buffer against psychological distress. Sociodemographic factors such as sex, marital status, cohabitation, and rural or urban background may shape these experiences, yet their combined influence remains insufficiently understood. This study examined how different forms of loneliness and life satisfaction relate to mental health in older adults, and how sociodemographic characteristics contribute to these associations.

**Methods:**

A cross‑sectional study was conducted with 143 community‑dwelling older adults aged 65 to 84 years living in urban and rural areas of La Rioja, Spain. Participants completed validated measures of life satisfaction; social, romantic, and family loneliness; depression; and anxiety. Logistic and linear regression models were used to identify predictors of depression and anxiety, and of life satisfaction and loneliness, respectively. When model assumptions were not met, correlation analyses were applied instead.

**Results:**

Social loneliness (*p*<0.001) and female sex (*p*=0.002) were significant risk factors for both depression and anxiety, whereas being 75 years or older (*p*=0.018) and higher life satisfaction (*p*<0.001) protected against depression. Romantic and social loneliness were strongly associated with lower life satisfaction (both *p*<0.001). Social loneliness increased among participants who cohabited (*p*=0.004), were socially isolated (*p*<0.001), and reported higher family loneliness (*p*<0.001), while life satisfaction acted as a protective factor (*p*<0.001). Romantic loneliness was lower among married participants (*p*<0.001), those raised in rural environments (*p*=0.003), and those reporting higher life satisfaction (*p*<0.001). Family loneliness correlated positively with social and romantic loneliness and negatively with life satisfaction (all *p*<0.01). Cohabiting and being married did not exert equivalent effects, indicating distinct relational influences.

**Conclusions:**

Life satisfaction and loneliness are key determinants of mental health in older adults, with sociodemographic factors shaping their expression. Social loneliness plays a central role in depression and anxiety, while marriage and rural upbringing protect against romantic loneliness. Interventions that enhance life satisfaction and address specific forms of loneliness may support psychological well‑being in later life.

**Supplementary Information:**

The online version contains supplementary material available at 10.1186/s12877-026-07671-9.

## Background

Human beings have a basic need to belong [1], which motivates the formation of interpersonal bonds. When these bonds cannot be established, individuals experience loneliness, understood as the subjective evaluation of actual and desired levels of satisfaction with one’s relationships [2]. Loneliness has been found to be associated with depression [3], poor executive functioning, sleep difficulties, and other physical and mental dysfunctions [4]. Older adults are particularly vulnerable to the negative effects of this condition [5].

However, loneliness is not a unitary construct, but a multidimensional experience that reflects deficits in different types of social relationships. Classical and contemporary models distinguish between social bonds that satisfy specific needs for the individual. Weiss [6] proposed that loneliness comprises two qualitatively distinct forms: the loneliness of emotional isolation, which arises from the absence of an attachment figure and resembles separation distress without an object, and the loneliness of social isolation, which results from the lack of a cohesive social network or sense of community. These forms emerge from different socioemotional dynamics, are activated under different conditions, and involve characteristic emotional responses. Weiss also emphasized that loneliness is a real emotional state rather than merely a cognitive appraisal of relational deficits. Contemporary models, such as those of De Jong Gierveld and Van Tilburg [7], converge with this view by distinguishing between emotional and social dimensions of loneliness. These experiences may be shaped by life circumstances such as marital status, living arrangements, and social participation.

Accumulating evidence suggests that loneliness in older adults is closely intertwined with mental health outcomes, particularly depression and anxiety. Longitudinal studies indicate that loneliness prospectively predicts both depression and anxiety in late life [8–10]. These associations appear to be moderated by sex and living conditions, among other variables [11].

Beyond psychopathology, life satisfaction, defined as the global cognitive evaluation individuals make regarding the quality of their own lives according to personally chosen standards [12–14], constitutes the central component of subjective well-being. Unlike the affective experience of loneliness, which reflects the subjective perception of having or lacking meaningful relationships, life satisfaction represents a broader and relatively stable judgment about one’s life trajectory. In older adults, higher life satisfaction has been associated with greater psychological resilience, more adaptive coping strategies, and better emotional regulation [14, 15]. Conversely, persistent loneliness has been linked to lower evaluations of life quality [16].

In older adulthood, life satisfaction and reduced loneliness emerge as key resources that support psychological adjustment. Maintaining a positive evaluation of one’s own life, together with the presence of meaningful social bonds, has been consistently associated with better coping in the face of age-related challenges and with more favorable mental health outcomes. These findings underscore the importance of examining how different dimensions of loneliness and life satisfaction interact to shape depression and anxiety in later life.

### Study purpose

On this basis, the present study was conducted with the purpose of confirming the hypothesis that higher life satisfaction and lower levels of loneliness are associated with lower levels of depression and anxiety in older adults. In addition, this study pursued two objectives: first, to determine whether life satisfaction and loneliness act as influencing factors in the presence of depression and anxiety in older adults; and second, to characterize the relationship between life satisfaction and loneliness within the context of partnership, family, and social relationships.

## Methods

### Study design

A paper-based cross-sectional study was conducted from October to December 2024 with older adults living in private homes across adjacent urban and rural areas of the Pyrenees in La Rioja, Spain. Rural areas were defined as settlements with less than 300 inhabitants/km² [17]. Older adults currently living in those areas were initially contacted through the Department of Health and Social Policies of La Rioja (Consejería de Salud y Políticas Sociales de La Rioja). Written informed consent was obtained from all participants prior of providing any personal information, and their participation was voluntary and anonymous. Data were collected in face-to-face interviews in community centres on dates previously agreed, following a protocol approved by the Comité de Ética de Investigación con medicamentos de La Rioja (CEImLAR), an independent ethics committee (Ref. CEImLAR-PI-795).

### Participants

Participants were required to be native Spanish speakers, aged 65 to 84 years, and residing in a private home in the La Rioja region to be eligible for inclusion. Individuals living in nursing homes, presenting cognitive impairment, or exhibiting total or severe physical dependency were excluded. The Mini Examen Cognoscitivo (MEC) [18], the Spanish adaptation of the MMSE [19], and the Barthel Index (BI) [20] were used as screening tests for cognitive impairment and dependency, respectively. Participants scoring below 25 on the MEC, indicating cognitive impairment, or below 61 on the BI, indicating physical dependency, were excluded.

### Main measures

#### Life satisfaction

It was measured with the Satisfaction With the Life scale (SWLS) [12]. The SWLS is composed by 5 items. Each one is answered following a Likert scale from 1 (strongly disagree) to 5 (strongly agree). A higher score indicates a greater overall agreement with the life.

#### Social loneliness

The social loneliness scale (ESTE-II) was used for this purpose. The ESTE-II (15 items) is a psychometric instrument specifically developed for measuring social loneliness in older adults [21]. The ESTE-II explores three aspects associated with social loneliness in older adults: perception of social support, participation in social activities, and familiarity with technologies devices that are currently used in social interactions (i.e. “do you use a computer?”, “do you use Internet?”). Items of the ESTE-II are answered using a 3-points frequency scale from 0 (always) to 2 (never). A higher score indicates a greater perception of social loneliness in older adults.

#### Romantic and family loneliness

The Short Social and Emotional Loneliness Scale (SELSA-S) was used for this purpose. The SELSA-S (15 items) is a multidimensional psychometric instrument designed to measure loneliness in adults [22]. The SELSA-S consists of three subscales assessing loneliness in social (5 items), family (5 items), and romantic (5 items) contexts. Developed on the basis of Weiss’ loneliness typology [2, 6], the SELSA-S can be applied either as a global measure of loneliness or as a domain-specific measure. Although, it was not originally developed for administration in older adults, it has demonstrated adequate psychometric properties in studies with this age group [23]. For this study, an adapted and validated version of the SELSA-S for older adults was used [24]. In the adapted version, items are rated on a simplified 7‑point scale ranging from “no” to “yes,” corresponding to the original “strongly disagree” to “strongly agree,” while retaining the same item wording as the original scale. Higher scores indicate greater perceived loneliness within each domain. For assessing romantic and family loneliness, the SELSA‑S/f and SELSA‑S/r were used, respectively.

#### Mental health

Anxiety and depression were used as two indicators of mental health deterioration. Symptoms related to both conditions were measured with the Geriatric Anxiety Inventory (GAI-20) and the Geriatric Depression Scale (GDS-15), respectively .The GAI-20 (20 items) is a short self-report or health professional-administered scale to explore anxiety symptoms [25]. The GAI-20 is a recognized screening tool, rather than a clinical diagnostic inventory, specifically designed to be administered in elderly people. The scale was initially developed to discriminate between those with and without any anxiety disorder and between those with and without Generalized Anxiety Disorder (according to DSM-IV criteria). Each item of the GAI-20 is rated as 0 (disagree) or 1 (agree), yielding a global score between 0 and 15. A higher global score indicates greater symptoms of anxiety. In addition, a global score higher than eight indicates anxiety. The GDS-15 (15 items) is another screening tool that was developed to identify symptoms of depression [26]. The GDS-15 can be also used as a self-report or a health professional-administered scale. Each item of the GDS-15 is rated as 0 (no) or 1 (yes), yielding a global score between 0 and 15. A score higher than five indicates depression.

#### Other measures

Jointly with the psychometric measures previously described, social isolation was assessed using a tool developed by the UK Biobank and applied in prior studies [27, 28]. This instrument comprises three dichotomous items addressing cohabitation, social contact, and participation in social activities, each scored 0 (no) or 1 (yes). A global score ranging from 0 to 3 was calculated, with values greater than 1 indicating social isolation. In addition, sociodemographic information was collected, including age, sex, marital status (married vs. other situations), cohabiting, having children, educational level (primary, secondary, or higher), and living environment (urban or rural) during childhood, adulthood, and at present.

### Statistical analysis

Psychometric reliability was calculated for all scales used. Psychometric reliability was estimated using Cronbach’s alpha and McDonald’s omega coefficients. Instruments with coefficients equal or higher than 0.70 were considered with an adequate internal consistency. Normality was estimated using Pearson’s chi-squared and Lilliefors-Kolmogorov-Smirnov tests.

Depression and anxiety scores were dichotomized to distinguish cases from non-cases. Each variable was analysed in a separate logistic regression model, using a backward stepwise regression procedure to select the most relevant predictors from the other variables collected. The models were designed to estimate the magnitude of the association between these indicators of mental health deterioration and the independent variables considered as explanatory factors. To assess the explanatory power of each logistic regression model, Nagelkerke’s R-squared was calculated. Finally, the strength of the association between the dependent variable and its explanatory variables was expressed using odds ratios.

Scores obtained in life satisfaction and loneliness measures were used as dependent variables in separate multiple linear regression analyses, with the other measures and sociodemographic variables collected as potential predictors. Measures of depression and anxiety were not included in these analyses. Each model was accepted only when it met the necessary conditions for statistical inference: normality, zero mean, constant variance, and uncorrelated residuals, in addition to linearity and absence of multicollinearity. To quantify the degree of practical significance of the models, the effect size (Cohen’s *f²*) was calculated. An effect size of 0.02 was interpreted as small, 0.15 as medium, and 0.35 as large [29].

## Results

### Participants and reliability

Of the 154 individuals initially contacted, seven were excluded in the screening tests and four due to age. This resulted in a final sample of 143 participants (124 female), with ages ranging from 65 to 84 years (M = 72, *SD* = 5.7). A full description is provided in Table 1.

**Table 1:** Demographic characteristics of the study sample (*n* = 143)

	Study sample
Sex	
Female	124 (87%)
Male	19 (13%)
Age	
Mean (*SD*)	72 (5.7)
Median [Min, Max]	71 [65, 84]
Marital status	
Currently married	61 (43%)
Widowed person	45 (31%)
Single person	16 (11%)
Other	21 (15%)
Having children	
No	25 (18%)
Yes	117 (82%)
Living environment (childhood)	
Urban settlement	42 (29%)
Rural settlement	101 (71%)
Living environment (adulthood)	
Urban settlement	66 (46%)
Rural settlement	77 (54%)
Living environment (old age)	
Urban settlement	42 (29%)
Rural settlement	101 (71%)
Cohabiting	
Yes	80 (56%)
No	63 (44%)
Education	
Primary	69 (48%)
Lower secondary	41 (29%)
Upper secondary	33 (23%)
Social isolation	
Socially isolated	16 (11%)
Non socially isolated	127(89%)

All administered psychometric instruments showed acceptable reliability, given McDonald’s coefficients higher than 0.70. The complete description of the scores of instruments and coefficients estimated is reported in Table 2. None of the instruments, however, met the assumption of normality.

**Table 2:** Descriptive analysis and reliability of scales administered

	*n*	PR	AR	Mdn	M (SD)	Alpha	Omega
Family loneliness (SELSA-S/f)	143	5–35	5–35	11	12(7)	0.69	0.84
Romantic loneliness (SELSA-S/r)	143	5–35	5–35	23	19(8)	0.65	0.88
Social loneliness (ESTE-II)	143	0–30	0–21	10	10(4)	0.70	0.78
Depression (GDS-15)	143	0–15	0–12	2	3(4)	0.86	0.89
Anxiety (GAI-20)	143	0–20	0–20	6	7(6)	0.93	0.94
Life satisfaction (SWLS)	143	5–25	5–25	18	17(5)	0.86	0.89

*n* study sample, *PR* Possible range, *AR* Actual range, *Mdn* Median, *M* Mean, *SD* Standard Deviation, *Alpha* Cronbach’s alpha coefficient, *Omega* McDonald’s omega coefficient

### Predictors of depression and anxiety

**Fig. 1:**
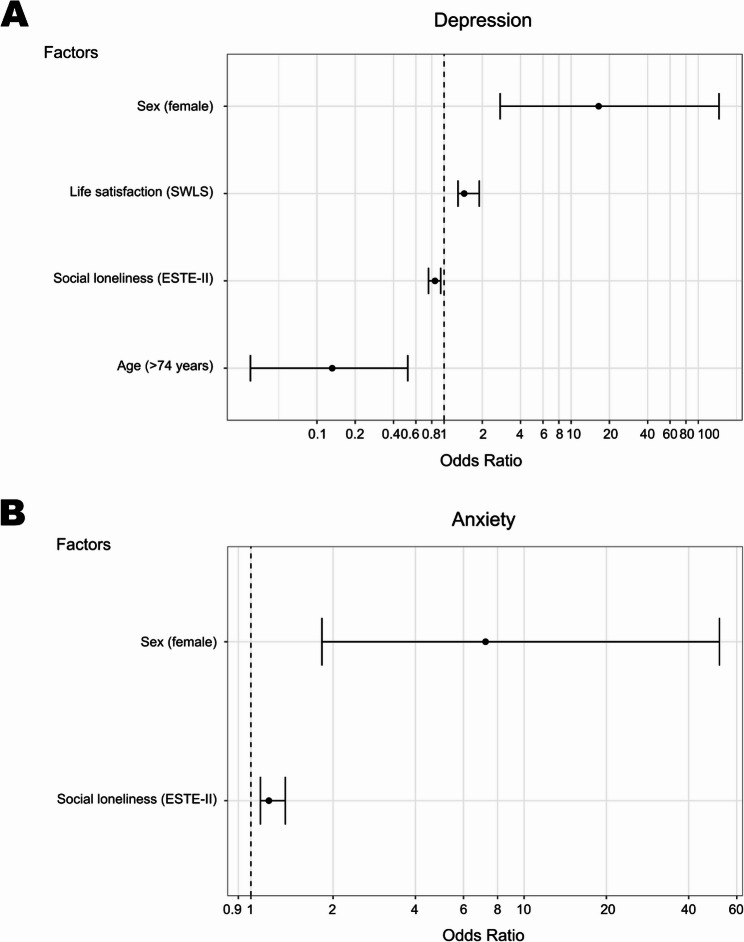
Odds ratio of explanatory factors of depression (**A**) and anxiety (**B**)

From all variables analyzed, female older adults and social loneliness appeared as risk factors for depression, while being 75 years or older and life satisfaction appeared as protective factors (Fig. 1A). The logistic model obtained showed good overall fit (Hosmer–Lemeshow χ²=2.48, *p* = 0.96), AIC = 104.7, and a Nagelkerke R² of 0.57. A summary of these findings is presented in Table 3. Female older adults and social loneliness emerged as risk factors also for anxiety, while none of the other variables studied reached statistical significance (Fig. 1B). The model obtained showed adequate fit (Hosmer–Lemeshow χ²=7.47, *p* = 0.49), AIC = 168.0, and a Nagelkerke R² of 0.17. A detailed summary of these findings is presented in Table 3.

**Table 3:** Logistic regression models for depression and anxiety

	β	SE	OR (95% CI)	*p*-value
Depression (GDS-15)Nagelkerke *R*^2^ = 0.57				
Age: >74 years	-1.96	0.72	0.14(0.03–0.52)	0.006
Sex: female	+ 2.87	1.00	17.6 (2.76–146)	0.004
Social loneliness (ESTE-II)	+ 0.42	0.09	1.54 (1.29–1.89)	< 0.001
Life satisfaction (SWLS)	-0.16	0.06	0.85 (0.75–0.94)	0.003
Anxiety (GAI-20) Nagelkerke R^2^ = 0.17				
Sex: female	+ 2.00	0.82	7.44 (1.82–51.8)	0.014
Social loneliness (ESTE-II)	+ 0.18	0.05	1.20 (1.08–1.34)	< 0.001

*β* logistic regression coefficient, *SE* Standard error, *CI* Confidence interval, *OR* Odds ratio, *p* *p*-Value

In both above mentioned models, sensitivity analyses indicated that excluding sex worsened the fit for depression (AIC = 112.4; R²=0.51) and anxiety (AIC = 174.6; R²=0.10), respectively. Given the imbalance between men and women in the study sample, a penalized logistic regression was also applied to assess the stability of the estimates. This approach yielded coefficients very similar to the standard models but with narrower confidence intervals, supporting the validity of including sex. A comparative summary of these findings, including models with sex, is provided in Supplementary Table 1.

### Relationship between life satisfaction and loneliness

Three linear regression models were obtained for life satisfaction, social loneliness, and romantic loneliness (see Table 4), all meeting the assumptions of normality, homoscedasticity, independence, multicollinearity, and linearity of residuals. No valid model was obtained for family loneliness, as the assumptions were not met. Therefore, a correlation analysis was conducted to examine its relationship with the other measures. This analysis showed a positive correlation between family loneliness and both social (ρ = 0.33, *p* < 0.001) and romantic loneliness (ρ = 0.32, *p* < 0.001), and a negative correlation with life satisfaction (ρ=-0.22, *p* = 0.006).

**Table 4:** Multiple regression models for life satisfaction, and for social and romantic loneliness

	β	SE	t	*p*-value
Life Satisfaction (SWLS)Adjusted *R*^2^ = 0.38				
Romantic loneliness (SELSA-S/r)	-0.26	0.05	-5.67	< 0.001
Social loneliness (ESTE-II)	-0.55	0.09	-6.01	< 0.001
Social loneliness (ESTE-II) Adjusted R^2^ = 0.38				
Cohabiting: yes	+ 1.30	0.54	2.40	0.017
Social isolation: yes	+ 3.90	0.85	4.60	< 0.001
Family loneliness (SELSA-S/f)	+ 0.13	0.04	3.17	0.0018
Life satisfaction (SWLS)	-0.31	0.05	-6.01	< 0.001
Romantic loneliness (SELSA-S/r) Adjusted R^2^ = 0.57				
Marital status: married	-9.70	0.96	-10.07	< 0.001
Childhood living environment: rural	-2.20	0.97	-2.29	0.024
Life satisfaction (SWLS)	-0.36	0.09	-4.03	< 0.001

*β* beta coefficient, *SE* Standard error, *t* t-experimental, *p* *p*-Value

The linear regression model for life satisfaction showed that both romantic and social loneliness were significantly associated with lower satisfaction with the life. The model explained a substantial proportion of variance (Adjusted R²=0.38; *F*_(2,140)_ = 44.6, *p* < 0.001, *f*^2^ = 0.64).

Regarding social loneliness, higher scores were associated with cohabiting, being socially isolated, and experiencing family loneliness. In contrast, life satisfaction acted as a protective factor. The model showed good explanatory capacity (Adjusted R^2^ = 0.38; *F*_(4,138)_ = 23.0; *p* < 0.001, *f*^2^ = 0.67).

Finally, a linear regression model was obtained for romantic loneliness explaining 57% of its variance (Adjusted R²=0.57; F_(3,139)_ = 64.2, *p* < 0.001, *f*^2^ = 1.38). According to the model, older adults who were married, those who had grown up in a rural area, and those reporting higher in life satisfaction presented significantly reduced romantic loneliness scores.

## Discussion

### Depression and anxiety

This study identified female sex and social loneliness as risk factors for depression in older adults, whereas being 75 years or older and reporting higher life satisfaction acted as protective factors. These findings align with previous evidence highlighting the relevance of psychosocial determinants in late‑life mental health [30]. The strong association observed for sex should be interpreted with caution given the imbalance between men and women in the sample; nevertheless, sensitivity analyses and penalized regression supported the validity of including sex, reinforcing the robustness of these findings. It is also important to note that the underrepresentation of men may reflect a broader pattern of lower engagement of older men in community‑based activities in the region, rather than a recruitment bias, which further contextualizes the observed imbalance. The protective role of advanced age may reflect selective survival or resilience mechanisms and a process of adjustment from active working life to a retired one [31], while life satisfaction appears to function as a key buffer against depressive symptoms [32]. However, it is also possible that poorer mental health, particularly depressive symptoms, may reduce perceived life satisfaction, which should be considered when interpreting this association [33].

For anxiety, female sex and social loneliness also emerged as significant risk factors, whereas none of the other variables analyzed reached statistical significance. Again, the effect of sex requires cautious interpretation due to sample imbalance, but sensitivity analyses and penalized regression confirmed its stability.

Taken together, these results underscore the influence of sex and social loneliness in understanding mental health outcomes in later life, with depression additionally shaped by protective influences of age and how older adults perceive their life.

### Relationship between life satisfaction and loneliness in older adults

The model for life satisfaction underscores the detrimental impact of specific dimensions of loneliness on well‑being in later life. Both romantic and social loneliness were strongly associated with lower life satisfaction, highlighting how the absence of emotionally fulfilling relationships and other psychosocial burdens can erode quality of life. These findings align with previous evidence showing that loneliness is not only a social phenomenon but also a key determinant of psychological resilience, life satisfaction, and even mortality risk [34–39]. The robustness of the model suggests that interventions aimed at reducing romantic loneliness and addressing related psychosocial stressors could play a crucial role in promoting well‑being among older adults.

The model for social loneliness highlights the relevance of psychosocial determinants in late‑life mental health. Social isolation, family loneliness, and cohabiting emerged as risk factors, whereas higher life satisfaction acted as a protective factor. The finding that older adults who did not cohabit reported lower scores in social loneliness is apparently counterintuitive, yet it may reflect the personal burden associated with cohabitation, such as caregiving responsibilities or family obligations that limit opportunities to socialize outside the household or couple environment. Although caregiving burden was not directly assessed in this study, previous research suggests that these responsibilities may restrict social engagement in later life [40–42]. In this sense, older adults living alone may benefit from two complementary mechanisms: on the one hand, having fewer family obligations can preserve greater autonomy and facilitate the maintenance of broader social networks; on the other hand, some authors have associated living alone with solitude, understood not as loneliness but as a voluntary and positive state of being alone, characterized by autonomy, self‑reflection, and emotional self‑sufficiency [43]. From this perspective, both the absence of family burden and the experience of chosen aloneness may jointly foster conditions that protect against social loneliness and support well‑being in later life.

The findings in romantic loneliness suggest that marriage is a strong protective factor against romantic loneliness in later life, consistent with previous studies highlighting the role of marital bonds in emotional well‑being among older adults [44] and other age groups [45]. Growing up in a rural environment was also associated with lower romantic loneliness, which may be explained by the presence of tighter community networks and stronger feelings of belonging, as described in research on social capital in rural contexts [46]. From the perspective of Weiss’s model of emotional loneliness [2, 6], this association may also be linked to the development of more stable attachment relationships across the life course. Rural environments often place greater emphasis on family life and long‑term partnerships, which may foster stronger feelings of emotional security and continuity from early family bonds to adult romantic relationships [46, 47]. By contrast, urban contexts may be characterized by higher relational mobility and greater challenges related to work–family reconciliation, potentially affecting the stability and perceived security of intimate bonds [4]. Although rural upbringing was directly assessed in the present study, the underlying relational and attachment‑related mechanisms were not directly measured; nevertheless, they provide a plausible theoretical framework for interpreting the protective role of a rural upbringing against romantic loneliness in later life. Finally, life satisfaction emerged as a key psychological resource, in line with literature emphasizing positive self‑perceptions as buffers against loneliness across its different dimensions. Recent evidence also supports this view [48], showing that higher life satisfaction mitigates the emotional impact of loneliness and contributes to psychological resilience in later life.

Taken together, these results emphasize that life satisfaction in older adults is highly sensitive to relational contexts, and that targeted strategies to foster meaningful connections may buffer against the adverse effects of loneliness. For example, preparatory workshops for individuals approaching retirement could help align expectations with the realities of later‑life social and relational contexts, while also supporting participants in identifying personal strengths and resources that may enhance their evaluations of life circumstances. In addition, structured group‑based activities designed to foster meaningful social engagement may help strengthen social networks during this transitional period.

## Conclusions

The present study highlights the central role of loneliness and life satisfaction in shaping mental health outcomes in older adults. Social loneliness emerged as a consistent risk factor for both depression and anxiety, whereas life satisfaction acted as a protective factor specifically against depressive symptoms. Romantic and social loneliness were also strongly associated with lower life satisfaction, underscoring the importance of relational contexts in subjective well‑being. Notably, the findings revealed that cohabiting and being married do not exert equivalent effects on loneliness, with marriage acting as a protective factor against romantic loneliness while cohabiting was associated with higher social loneliness. This distinction cautions against assuming that living with someone and being in a marital relationship provide the same relational benefits. Overall, the results reinforce the multidimensional nature of loneliness and demonstrate that different relational domains contribute uniquely to psychological adjustment in later life. Promoting meaningful social connections and enhancing older adults’ evaluations of their own lives may therefore be key strategies for mitigating mental health deterioration in aging populations.

### Limitations

This study presents several limitations that should be considered when interpreting the findings. Its cross‑sectional design prevents establishing causal relationships between loneliness, life satisfaction, and mental health outcomes. The sample was predominantly composed of women, which may limit the generalizability of sex‑related findings, despite the robustness checks performed using penalized regression models. This imbalance may be partly explained by a broader pattern of lower engagement of older men in community‑based activities in the region, rather than by differential willingness to participate; however, it remains an important consideration when interpreting sex differences. The reliance on self‑report measures may introduce response biases, particularly in constructs such as loneliness and life satisfaction. The study also did not assess physical health conditions or subclinical forms of functional deterioration beyond the exclusion criteria, which may influence both the experience of loneliness and the evaluation of life satisfaction in older adults. In addition, given the age of the participants, gendered cultural norms and socialisation experiences characteristic of this cohort may have shaped how women in this generation perceive partnered life, family roles, and social relationships, which could influence their interpretations of emotional needs related to different loneliness typologies. Furthermore, although rural upbringing was included as a variable in the analyses, the broader contextual influence of living in predominantly rural environments throughout the life course was not directly assessed, and such contextual factors may shape social expectations and the perception of loneliness. Moreover, the study did not include life‑course or retrospective measures, which limits the ability to account for earlier experiences or coping competencies that may shape how loneliness is perceived in later life. Finally, the study was conducted in a specific geographical region, and cultural or contextual factors may influence the observed patterns.

### Future research

Future research should employ longitudinal designs to clarify the temporal and causal pathways linking loneliness, life satisfaction, and mental health in older adults. It would be valuable to explore the mechanisms through which different forms of loneliness exert their influence, including the role of social cognition, emotion regulation, and perceived social support. Given the relevance of relational contexts, future studies could examine how transitions in partnership, family dynamics, or community engagement shape loneliness trajectories over time. In this context, future research should explicitly assess caregiving roles and perceived caregiving burden to clarify their contribution to social loneliness, particularly among older adults who cohabit. Developing and validating instruments specifically tailored to assess romantic and family loneliness in older adults would strengthen the precision of future analyses. Future research could also incorporate additional components of subjective well‑being, such as affective dimensions, to provide a more comprehensive understanding of psychological adjustment in later life. In addition, future studies should consider recruitment strategies specifically tailored to older men, who may be less likely to participate in community‑based initiatives, to achieve more balanced samples. Finally, intervention studies targeting both loneliness reduction and the enhancement of life satisfaction could provide actionable insights for promoting psychological well‑being in aging populations.

## Supplementary Information

Supplementary Material 1. Supplementary Table 1. Comparison of coefficients between logistic regression (LRM) and penalized logistic regression (pLRM) models for symptoms of depression.

Supplementary Material 2. Supplementary Table 2. Comparison of coefficients between logistic regression (LRM) and penalized logistic regression (pLRM) models for symptoms of anxiety.

## Data Availability

Data supporting the findings of this study are available from the corresponding author upon reasonable request.

## Abbreviations

AIC: Akaike Information Criterion
ESTE-II: Social Loneliness Scale
GAI-20: Geriatric Anxiety Inventory
GDS-15: Geriatric Depression Scale
MEC: Mini Examen Cognoscitivo
MMSE: Mini Mental State Examination
SELSA-S: Social and Emotional Loneliness Scale for Adults
SWLS: Satisfaction with the Life Scale
