# Supplementary material for: Life satisfaction, loneliness, and mental health in older adults: sociodemographic influences in a cross-sectional analysis
Source: BMC Geriatr. 2026 May 20;26:946. doi: 10.1186/s12877-026-07671-9 (PMC13360845; doi:10.1186/s12877-026-07671-9)
Supplement: Supplementary file 1 — Supplementary Material 1. Supplementary Table 1. Comparison of coefficients between logistic regression (LRM) and penalized logistic regression (pLRM) models for symptoms of depression. [file 12877_2026_7671_MOESM1_ESM.docx]

**Supplementary Table 1.** Comparison of coefficients between logistic regression (LRM) and penalized logistic regression (pLRM) models for symptoms of depression

| **Factors** | **LRM** | | | **pLRM** | | |
| --- | --- | --- | --- | --- | --- | --- |
|  | **β** | ***SE*** | ***p-*value** | **β** | ***SE*** | ***p-*value** |
| Age: >74 years | -1.96 | 0.72 | 0.006 | -1.80 | 0.66 | <0.001 |
| Sex: female | +2.87 | 1.00 | 0.004 | +2.63 | 0.92 | <0.001 |
| Social loneliness (ESTE-II) | +0.42 | 0.09 | <0.001 | +0.39 | 0.09 | <0.001 |
| Life satisfaction (SWLS) | -0.16 | 0.06 | 0.003 | -0.15 | 0.05 | <0.001 |

β, beta coefficient; *SE*, standard error; *p*, p-Value
