# Supplementary material for: Life satisfaction, loneliness, and mental health in older adults: sociodemographic influences in a cross-sectional analysis
Source: BMC Geriatr. 2026 May 20;26:946. doi: 10.1186/s12877-026-07671-9 (PMC13360845; doi:10.1186/s12877-026-07671-9)
Supplement: Supplementary file 2 — Supplementary Material 2. Supplementary Table 2. Comparison of coefficients between logistic regression (LRM) and penalized logistic regression (pLRM) models for symptoms of anxiety. [file 12877_2026_7671_MOESM2_ESM.docx]

**Supplementary Table 2.** Comparison of coefficients between logistic regression (LRM) and penalized logistic regression (pLRM) models for symptoms of anxiety

| **Factors** | **LRM** | | | **pLRM** | | |
| --- | --- | --- | --- | --- | --- | --- |
|  | **β** | ***SE*** | ***p-*value** | **β** | ***SE*** | ***p-*value** |
| Sex: female | +2.00 | 0.82 | 0.014 | +1.79 | 0.74 | <0.001 |
| Social loneliness (ESTE-II) | +0.18 | 0.05 | <0.001 | +0.17 | 0.05 | <0.001 |

β, beta coefficient; *SE*, standard error; *p*, p-Value
